# Using Conversational AI to Facilitate Mental Health Assessments and Improve Clinical Efficiency Within Psychotherapy Services: Real-World Observational Study

**DOI:** 10.2196/44358

**Published:** 2023-12-13

**Authors:** Max Rollwage, Johanna Habicht, Keno Juechems, Ben Carrington, Sruthi Viswanathan, Mona Stylianou, Tobias U Hauser, Ross Harper

**Affiliations:** 1 Limbic Limited London United Kingdom; 2 Everyturn Mental Health Gosforth United Kingdom; 3 Max Planck University College London Centre for Computational Psychiatry and Ageing Research University College London London United Kingdom; 4 Department of Psychiatry and Psychotherapy Medical School and University Hospital Eberhard Karls University of Tübingen Tubingen Germany; 5 German Center for Mental Health (DZPG) Tubingen Germany

**Keywords:** artificial intelligence, National Health Service, NHS, Improving Access to Psychological Therapies, IAPT, mental health, mental health assessment, triage, decision-support, referral, chatbot, psychotherapy, conversational agent, assessment, Talking Therapies

## Abstract

**Background:**

Most mental health care providers face the challenge of increased demand for psychotherapy in the absence of increased funding or staffing. To overcome this supply-demand imbalance, care providers must increase the efficiency of service delivery.

**Objective:**

In this study, we examined whether artificial intelligence (AI)–enabled digital solutions can help mental health care practitioners to use their time more efficiently, and thus reduce strain on services and improve patient outcomes.

**Methods:**

In this study, we focused on the use of an AI solution (Limbic Access) to support initial patient referral and clinical assessment within the UK’s National Health Service. Data were collected from 9 Talking Therapies services across England, comprising 64,862 patients.

**Results:**

We showed that the use of this AI solution improves clinical efficiency by reducing the time clinicians spend on mental health assessments. Furthermore, we found improved outcomes for patients using the AI solution in several key metrics, such as reduced wait times, reduced dropout rates, improved allocation to appropriate treatment pathways, and, most importantly, improved recovery rates. When investigating the mechanism by which the AI solution achieved these improvements, we found that the provision of clinically relevant information ahead of clinical assessment was critical for these observed effects.

**Conclusions:**

Our results emphasize the utility of using AI solutions to support the mental health workforce, further highlighting the potential of AI solutions to increase the efficiency of care delivery and improve clinical outcomes for patients.

## Introduction

### Background

Common mental illnesses have become the leading cause of disability worldwide [1]. Access to high-quality mental health care is therefore crucial, with up to 25% of the population experiencing depression or anxiety disorders [2,3]. The COVID-19 pandemic has further highlighted the need for accessible mental health treatment, precipitating increased cases of anxiety, depression, and other mental health symptoms [4-9]. Addressing this high demand is challenging for many mental health services that already struggle to provide adequate treatment with limited resources, resulting in impaired patient experience and, ultimately, worse treatment outcomes [10].

One particular challenge that mental health services face is the long wait time between the point from when a patient seeks support and when they begin treatment. For instance, in the English National Health Service (NHS), between 2021 and 2022, 31% of referrals to Talking Therapy services dropped off the wait list before starting treatment, and 9% of patients waited for >6 weeks for their clinical assessment [11]. In addition, a further 47% of patients experienced *hidden waits* of >28 days between clinical assessment and their first treatment session, contrary to the guidance from the National Institute of Health and Care Excellence, which highlights the importance of timely access to treatment [12].

Notably, against the backdrop of rising referrals, the needs of patients are unlikely to be addressed through an increase in the clinical workforce; in fact, there exists a national shortage of qualified staff [13]. To remedy this precarious situation, it has been repeatedly suggested that digital tools might represent a viable opportunity to improve the efficiency and quality of service delivery, as well as to enhance patient outcomes and experience [14-17].

Previous studies have explored the use of digital solutions in health care settings, such as artificial intelligence (AI)–based interventions and conversational agents. However, these studies have mainly focused on treatment support or remote monitoring [18]. Moreover, there is little evidence of the efficacy of such tools in real-world clinical settings [18,19]. Within the field of mental health care, the use of AI and conversational agents has mainly focused on self-care tools [20], whereas the efficacy of AI in supporting clinicians in their delivery of high-quality care has not been explored. The use of AI is well suited to address the supply-side issues faced by mental health care providers by improving the allocation of staff time to boost service capacity through the support and augmentation of clinicians [21,22]. For example, AI can enable health care professionals to prioritize tasks and streamline processes by automating low-level clinical functions such as adaptive information gathering to inform assessment or treatment sessions conducted by a trained clinician.

Digital innovation to support referral and clinical assessment is earmarked as a key area to increase service capacity within mental health care. One of the main aims of the referral process is to collect information that can be used for clinical assessment to identify symptoms and triage patients into the appropriate treatment pathways. Therefore, the referral process and clinical assessments represent promising targets for automation. These early parts of the care pathway are typically conducted by trained mental health professionals and require considerable time from these overburdened clinical staff. Indeed, studies have found that NHS Talking Therapies (previously Improving Access to Psychological Therapies (IAPT)) services spend up to 25% of their annual budget on clinical assessments [23]. Automation in this area represents a viable opportunity to release clinical time and resources that can be reallocated to other stages of the care pathway.

In addition to service efficiency, other patient benefits can be generated through the implementation of AI-enabled digital solutions. Direct benefits include reduced barriers to entry, such as social stigma and time constraints [24], resulting in a more accessible and patient-focused referral process. In addition, previous research suggests that patients are more likely to report severe symptoms in digital solutions [25], which can lead to more accurate referral information. As a result, clinicians receive a more comprehensive overview of the problems faced by their patients. This presents an opportunity to accelerate clinical assessment, improve pathway allocation, and spend more time during clinical contacts to focus on building a strong relationship with the patient. Indirectly, increased overall efficiency of the service will free up resources that can be reallocated to increase the number of available treatment sessions, which is known to improve clinical outcomes [26].

Therefore, we hypothesize that the use of an AI-enabled referral tool compared with other means of referral will reduce assessment times, reduce wait times for assessment and treatment, reduce dropout rates, reduce changes in treatment allocation, and improve recovery rates. Moreover, we hypothesize that these effects should be largely driven by the collection of clinically relevant information, which can provide valuable context for clinicians at assessment.

### Objectives

In this study, we evaluated the impact of an AI self-referral tool, a conversational AI chatbot (Limbic Access [Limbic Limited]), in a real-world scenario. This AI self-referral tool is already implemented as part of routine care across multiple NHS Talking Therapy services in England. We analyzed data from 1 service provider with Talking Therapy services across England. Data were collected from 64,862 patients who were referred for care either via the AI self-referral tool or via alternative methods of referral. We show that the AI solution improves clinical efficiency, reduces wait times and dropout rates, provides more accurate treatment allocation, and increases recovery rates. We further show that frontloading the collection of clinically relevant information ahead of the clinical assessment is a major driver for these observed improvements. Therefore, our findings provide novel empirical evidence that mental health care can be significantly improved through AI solutions that support trained clinicians in their daily work.

## Methods

### AI Self-Referral Tool

In this study, we evaluated the effects of a novel AI self-referral tool (Limbic Access), which was implemented as part of routine mental health care in several NHS Talking Therapy services. Limbic Access is a commercial product and was developed and commercialized by some of the authors in collaboration with NHS Talking Therapy services. This tool was initially tested in a pilot study with a sample of 7176 patients with 1 NHS Talking Therapy provider. After the successful completion of this pilot study, the tool was rolled out commercially across multiple NHS Talking Therapy providers.

This self-referral tool is a conversational chatbot integrated into the service’s website and assists patients in making a referral by collecting the necessary intake information as required by the Talking Therapy program (eg, eligibility criteria, contact details, and demographic information). Furthermore, the chatbot collects additional clinical information about the patient’s presenting symptoms, such as the Patient Health Questionnaire-9 (PHQ-9) [27], Generalized Anxiety Disorder Assessment-7 [28], Work and Social Adjustment Scale [29], and a selection of additional screening questions. These routine outcome measures and screening questions are typically not collected at the point of referral in NHS Talking Therapies. All the information collected by the AI self-referral tool is then attached to the referral record within the Talking Therapy service’s electronic health record to support clinicians in preparing a high-quality and high-efficiency clinical assessment.

**Figure 1:**
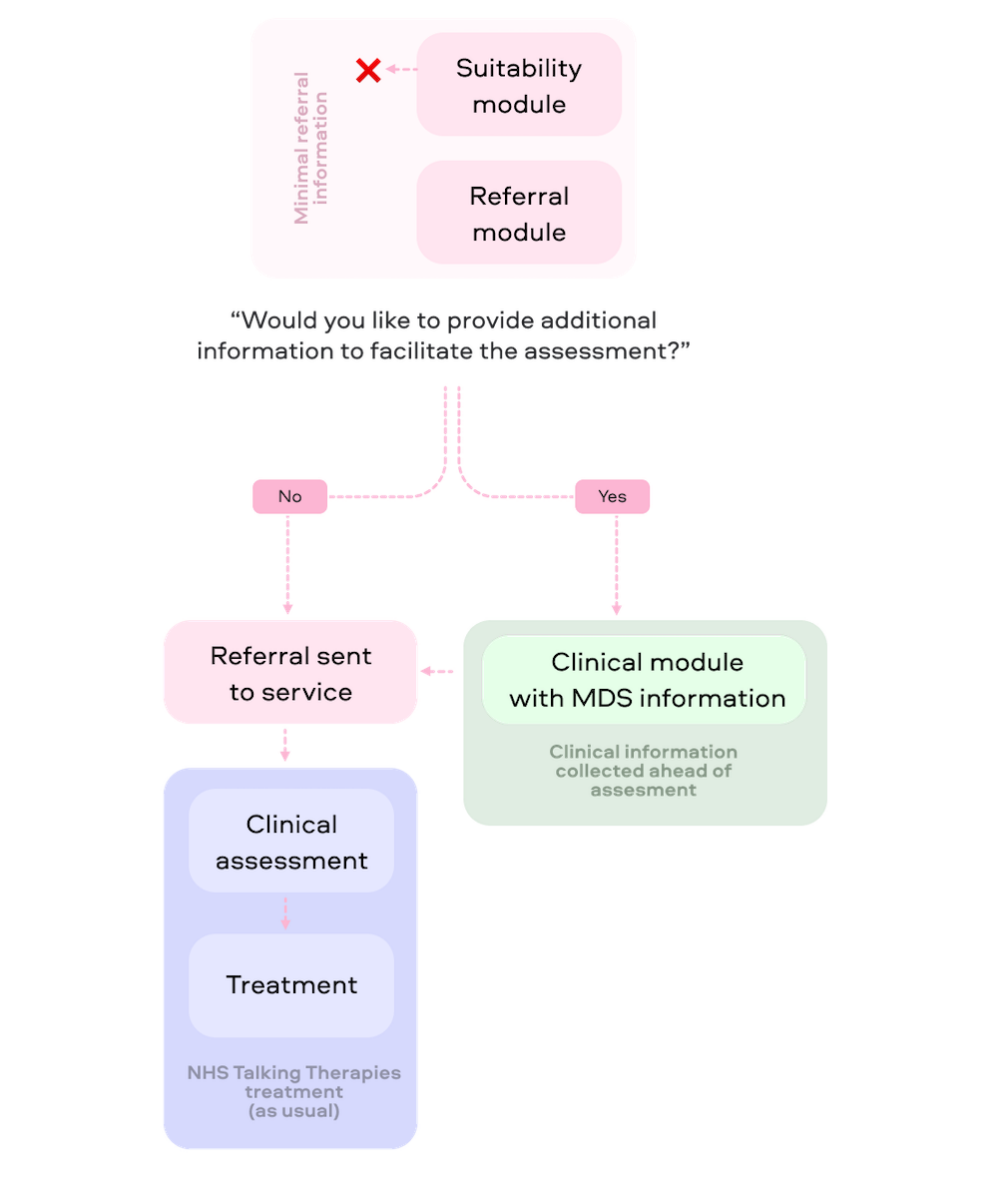
Pathway of the AI self-referral tool. The tool is embedded on National Health Service (NHS) Talking Therapies service’s web page and pops up when a potential patient navigates to that page. Upon initiating an interaction with the chatbot, the eligibility of the patient is determined in the eligibility module. If ineligible, the patient is signposted out of the service (indicated with a red cross mark). The signposting is based on the same standard characteristics that would be applied in other referral pathways, such as patient’s location and age, to ensure that only patients from the service’s catchment area and patients who are suited in terms of age will be referred. This ensures that patients do not complete the whole referral process to then be signposted elsewhere later on. Signposting out is unrelated to their mental health symptoms. The eligible patient then continues through the referral module which produces the minimal data set needed to refer the patient to the Talking Therapies service. After the referral module, the patient is asked whether they would like to provide additional information. If they consent, they fill in additional information regarding their mental health issues, which is added to the referral record sent to the Talking Therapies service. If they disagree, their referral is sent directly to the service. MDS: minimum data set.

It is important to note that when guiding a patient through referral to Talking Therapies, the AI tool uses a *checkpoint*, where there exists a point at which the patient has provided minimal information required to submit a referral. At this checkpoint, all the required information to submit the patient’s referrals to the service was collected. However, patients were then asked whether they would like to provide additional *clinical* information regarding their mental health issues, which was specifically designed to facilitate a clinician-led assessment (Figure 1). This additional information includes free-text input regarding the patient’s presenting symptoms as well as standardized, clinically validated routine outcome measures and screening questions. Empirically, most patients choose to provide additional information (approximately 97% of referrals); however, a subset of patients only provided minimally required information at referral (approximately 3% of referrals). This allowed us to implement a quasi-experimental design to test the effects of collecting clinical information on patient treatment outcomes.

### Clinical Implementation of the AI Self-Referral Tool

To derive maximal clinical value from an AI self-referral tool, appropriate implementation of this tool within a wider service environment is of critical importance. Indeed, the realized benefits of any digital tool rely on how it is used in practice.

Within the evaluated psychotherapy service (Everyturn Mental Health), clinical information collected by the AI self-referral tool was used to triage the severity of patient case presentations (eg, mild, moderate, and severe cases of depression can be differentiated based on the magnitude of the PHQ-9 score). Case presentation, symptom severity, and any associated risk factors are then used by the service to schedule an appropriate duration for a clinician-led assessment (ie, complex or severe cases require longer assessment slots and simpler or mild cases may only require shorter assessment slots). In this way, the NHS Talking Therapy service can use the clinical information to allocate clinical resources in a tailored and efficient manner.

The psychotherapy service additionally enabled a “direct booking” feature within the AI self-referral tool, which provided a means for patients to directly book a preferred time for their clinician-led assessment in the service’s calendar, thus reducing the administrative burden on the service and enabling faster access to a clinical assessment. This might be one mechanism by which this novel referral pathway could reduce wait time for patients.

Finally, all clinical information collected in the AI self-referral tool is programmatically transferred to the service’s chosen patient management system, which can be accessed by the clinician leading the clinical assessment. This provides support to the reviewing clinicians with richer contextual information.

We believe that these implementation decisions for an AI self-referral tool are crucial to consider with respect to the expected effects on service efficiency and quality of care.

### Design

Real-world data were collected from patients entering and receiving mental health care treatment through one specific provider of NHS Talking Therapy services (Everyturn Mental Health) between November 2021 and August 2022. The participating mental health services comprised 9 individual Talking Therapy services in different regions throughout England. This allowed us to include data from patients representing diverse geographic and demographic backgrounds (refer to Multimedia Appendix 1 for details on the demographic characteristics of the sample).

In this study, we examined the between- and within-group effects of this AI self-referral solution. In the between-group context, we compared patients who referred themselves to Talking Therapy services through the AI tool with those who were referred through other methods (eg, telephone referrals, referrals via a web form, general practitioner referrals, and referrals via other primary health care services). A comparison of these 2 groups was made possible because of the constant availability of alternative self-referral methods alongside the AI self-referral tool. Overall, these data comprised 64,862 patients, of whom 21,568 (33.25%) patients were referred through the AI self-referral tool and 43,294 (66.75%) patients were referred through alternative routes.

In the within-group context, we compared users referring through the AI self-referral tool who also completed the full clinical information (clinical information group: 20,860/21,546, 96.82% patients) with those who only completed the minimally required information for a referral (no clinical information group: 686/21,546, 3.18% patients). This allowed for a comparison of the effects of providing clinical information ahead of the assessment to evaluate some of the mechanisms by which the AI self-referral tool achieved its effects. Minimal referral information was defined as patients not completing all relevant clinical information asked for in the self-referral process. It was expected that only a small proportion of patients would not provide complete clinical information, as the AI self-referral tool was designed to increase engagement and ensure that a maximum number of patients complete all relevant information ahead of the clinical assessment.

### Ethical Considerations

As determined by the NHS and in accordance with National Institute of Health and Clinical Excellence principles [30], clinical audit studies within the NHS Talking Therapy framework do not require additional patient consent or ethical approval [30]. Moreover, the study team received written confirmation from the Health Research Authority of England that this study constitutes a service evaluation and, therefore, did not require additional ethical approval. When registering to use the AI self-referral tool, patients provided written informed consent as part of a privacy policy agreement, allowing the service to use anonymized patient data for auditing purposes and to support research.

### Outcome Measures

The outcome measures reported in this study were assessed routinely during mental health care delivered by NHS Talking Therapy services. Anonymous data were publicly reported on the NHS Digital website [31] for the evaluation of NHS Talking Therapy services performance. Therefore, no additional data beyond routine care data were collected for this study.

### Assessment Duration

We evaluated whether the use of the AI self-referral tool improved clinical efficiency by reducing the time required to complete a high-quality clinical assessment. The required length of clinical assessment was measured in minutes.

### Wait Time for Clinical Assessment

We evaluated whether the use of the AI self-referral tool reduced the wait time for clinical assessment. The required wait time for clinical assessment was measured in days, from the day of referral to the day of the clinical assessment.

### Wait Time for Treatment

We evaluated whether the use of the AI self-referral tool reduced the wait time to the start of treatment. The wait time for treatment was measured in days, from the day of referral to the day of the first treatment session. Only the data of patients who entered treatment were used for this analysis because, for some patients in the clinical assessment, it might be decided that no treatment is required.

### Dropout Rate

We determined whether the use of the AI self-referral tool would reduce the likelihood of patients dropping out of the service at any point during the care pathway. Dropouts were defined as those patients who canceled an appointment and did not rebook a new appointment. The dropout rate was measured as the percentage of patients who dropped out of the treatment.

### Change in Allocated Treatment Level

We evaluated whether the use of the AI self-referral tool would enable more accurate clinical assessment. A more accurate clinical assessment would manifest in patients assigned to the appropriate treatment pathway; therefore, the treatment pathway would be less likely to change during treatment. Changes in treatment are known as stepups and stepdowns in NHS Talking Therapies. We measured the accuracy of treatment allocation as the percentage of patients whose treatment was stepped up or down. Only data from patients who received and finished treatment were used for this analysis because the accuracy of treatment allocation can only be assessed after the treatment ends.

### Recovery Rate

We evaluated whether the use of the AI self-referral tool would enable a higher rate of recovery in the Talking Therapy service. The recovery of patients is assessed at the end of treatment, and the definition of reliable recovery is systematically used in NHS Talking Therapy services [32]. This was measured by administering an appropriate disorder-specific outcome questionnaire and was defined as a significant reduction in symptom scores (ie, PHQ-9 score: improved by at least 6 points and Generalized Anxiety Disorder Assessment-7 score: improved by at least 4 points) from the beginning to the end of treatment and a score below the clinical cut-off at the end of treatment. We measured the recovery rate as the percentage of patients who achieved reliable recovery. Only data from patients who received and finished treatment were used for this analysis because reliable recovery could only be assessed after completion of the treatment.

### Analysis

For the analysis of wait time for treatment, we only analyzed data from patients who had entered treatment. We included patients who had finished their treatment for the changes in treatment allocation and recovery rate analyses.

Because this was not a randomized controlled trial, there may have been differences in the characteristics of the patients referring through the AI tool versus the standard pathway, as well as *within* the AI self-referral tool cohort between patients with clinical information and patients without clinical information. Therefore, we statistically controlled for these potential differences to ensure that our observed results could not be explained by these confounding factors.

The confounding factor of main concern was the severity of patients’ mental health symptoms. These data were included for every patient, allowing us to control for this confounding factor when comparing the AI tool and standard referral pathways. We measured severity as the step of treatment level that patients were assigned to and controlled for severity in any analysis we conducted.

There was only limited information about the group of patients with other referral pathways available to ensure the anonymity of this group. No demographic information or any personally identifiable information was provided for these patients to ensure complete anonymity of data. Therefore, we were unable to control for demographic differences or any other personal information in this data group.

Demographic information was available for patients who were referred through the AI tool. Therefore, for comparison of patients who did and did not provide complete clinical information (all referred via the AI self-referral tool), all analyses controlled for a list of demographic variables (eg, age, gender, ethnicity, disability status, and receiving previous mental health support).

To adequately control for the above-mentioned covariates, we constructed multiple linear regression models for continuous outcome measures and multiple logistic regression models for binary outcome measures. The group was used as a predictor variable (AI vs standard referral comparison: 0=standard referral and 1=AI self-referral; clinical information vs no clinical information comparison: 0=no clinical information and 1=clinical information), and severity was included as a covariate. For the clinical assessment time, wait time to clinical assessment, wait time for treatment, and severity and demographics were the only potentially confounding effects that we controlled for.

Regarding dropout rates, it is possible that increased assessment and wait times could have indirectly led to increased dropouts. Therefore, we controlled for severity and demographics, assessment, and wait time as covariates in the logistic regression model to predict the dropout rates. This analysis will reveal whether the effects on dropout rates are completely explained by the changes in assessment and wait time or whether the use of the AI self-referral tool has an additional and independent effect on dropout rates.

Changes in treatment allocation could potentially be influenced by all the factors mentioned above, including dropout rates. Therefore, we controlled for severity and demographics, dropout rates, assessment, and treatment times in the logistic regression to predict changes in treatment allocation.

Finally, the recovery rate is the last measure of interest, which, in principle, could be influenced by all the factors mentioned above. In particular, changes in treatment allocation (ie, accuracy with which treatment allocation was assigned) could potentially explain why differences in recovery rates were observed. To evaluate whether the effects on the recovery rate could be explained by effects on these other variables or whether it represented independent and additional effects of the AI solution, we included severity and demographics, assessment time, wait time, dropout rates, and changes in treatment allocation as covariates in the logistic regression predicting recovery rates.

### Qualitative Analysis on the Reasons to Provide Clinical Information

To investigate the impact of clinical information on relevant outcome measures, we compared patients who provided clinically relevant information to those who did not provide this information.

Because this comparison was a quasi-experimental setup (ie, patients were not randomized into the conditions), we aimed to understand in more detail why patients chose to provide or not provide clinical information.

For this purpose, we analyzed qualitative data from a previous user experience study (unpublished) in which 32 ex-patients tested the AI self-referral tool and answered a subsequent survey on their experience with it. The original focus of this study was to identify potential weaknesses in the design of the AI self-referral tool, thus emphasizing ways to improve the product. This survey included the user experience questionnaire [33] and qualitative feedback questions about their experience. For this purpose, in this study, we focused on the qualitative feedback of the users. We first performed reflexive thematic analysis on feedback entries [34], with 2 of the authors open-coding all the feedback samples. The initial codes were discussed with the larger group of authors, and a consensus was reached on the resulting themes after 2 meetings. The list of resulting themes included comprehension, information about the further steps, ease of user interface use, number of questions, *heavy* nature of questions, ease of access, and the advantages of the tool’s human-free nature. Finally, 2 researchers coded each feedback sample with one of the themes, and the frequency of each category was analyzed. Therefore, we specifically focused on the frequency of the number of questions and the “heavy” nature of questions themes because these were related to the collection of clinically relevant information.

## Results

### Between-Group Results: Patient Referrals Made via the AI Tool Versus Alternative Routes

We first tested whether the groups of patients were comparable in terms of their severity of mental health conditions. The groups differed in their severity (Mann-Whitney *U* test; *P*<.001). Patients referred through the AI self-referral tool showed slightly lower severity (mean step of care=1.5) than those referred through other pathways (mean step of care=1.69). Although this was expected based on anecdotal evidence that patients referred through standard pathways show higher severity than patients referred through the AI tool, this finding indicates that it is critical to control for severity in the subsequent analyses.

### Assessment Time

A major aspect of an AI self-referral tool is the clinical efficiency generated through this product by reducing the time needed for a clinical assessment. Indeed, in the AI group (mean assessment time=41.6 min), the clinical assessment required, on average, 12.7 minutes less time (Multimedia Appendix 2) compared with the standard referral pathway group (mean assessment time 54.4 minutes). This effect was statistically significant (*t*_64,861_=−116.57; *P*<.001) and could not be explained by differences in severity because the effect remained significant after controlling for this factor (*P*<.001). This finding indicates that the use of AI in the self-referral process creates clinical efficiency by reducing clinical assessment times.

### Wait Time for Clinical Assessment

Then, we investigated whether the AI self-referral tool affected the time that patients had to wait for their clinical assessment. Indeed, in the AI group, the wait time for a clinical assessment was shorter (mean 15.2 days; Multimedia Appendix 2) than that for the standard referral pathway group (mean 17.4 days). This effect represented an average reduction in wait time of 2.2 days and was statistically significant (*t*_64,861_=−14.66; *P*<.001). This effect could not be explained by differences in severity because the effect remained significant after controlling for this factor (*P*<.001). This finding indicates that the AI tool reduced the wait times for clinical assessments.

### Wait Time to Treatment

Further, we investigated whether the AI self-referral tool affected the time patients had to wait until the first treatment session. In the AI group, the wait time for the first treatment session was shorter (mean 75.6 days; Multimedia Appendix 2) than that for the standard referral pathway group (mean 80.6 days). This effect represented an average reduction in wait time of 5 days and was statistically significant (*t*_33,269_=−7.1; *P*<.001). This effect could not be explained by differences in severity because the effect remained significant after controlling for this factor (*P*<.001). This finding indicates that the AI tool reduced wait time for accessing mental health treatment.

### Dropout Rate

Then, we investigated whether the AI self-referral tool affected the probability of patients dropping out of the treatment. The probability of dropping out of treatment was significantly reduced (*t*_33,269_=9.03; *P*<.001) from 26.7% probability in the standard referral pathway group to 21.9% probability in the AI tool group (Multimedia Appendix 2). This effect could not be explained by differences in severity or assessment and wait times because the effect remained significant after controlling for this effect (*P*<.001). This finding indicates that the use of the AI tool in the self-referral process reduced the likelihood of patients dropping out of the treatment pathway.

### Change in Allocated Treatment Level

Subsequently, we investigated whether the AI self-referral tool affected the accuracy of clinical assessment by investigating the effects on the changes in treatment allocation (ie, the lower rate of change equals improved accuracy of clinical assessment). Changes in treatment allocation were significantly reduced (*t*_20,317_=−8.290; *P*<.001) from 10.5% of patients receiving a change in treatment in the standard referral pathway group to 5.8% in the AI tool group (Multimedia Appendix 2). This effect could not be explained by differences in severity, dropout rates, or assessment or wait times because the effect remained significant after controlling for these factors (*P*<.001). This finding indicates that the AI self-referral tool improved clinical assessment accuracy, thus requiring fewer changes in allocation during treatment.

### Recovery Rates

Finally, we investigated whether the AI self-referral tool affected the recovery rates of patients. Indeed, in the AI group (recovery rate=58%), the recovery rates were significantly higher (*t*_20,317_=38.7; *P*<.001; Multimedia Appendix 2) than those in the standard referral pathway group (recovery rate=27.4%). The effect size is noteworthy as the recovery rate was twice as high in the AI group compared with the standard referral pathway group. This effect could not be explained by differences in severity, dropout rates, assessment and wait times, or by changes in treatment allocation because the effect remained significant after controlling for these factors (*P*<.001). This finding indicates that the use of AI tool in the referral process improved the recovery rates of patients referred through this tool in addition to the other effects presented in this study.

### Within-Group Results: Effect of Additional Clinical Information Collected Ahead of Clinician-Led Assessment

Having established the effects of referring through an AI self-referral tool compared with other methods of referral, we investigated more closely the mechanism through which these improvements were achieved. Our initial hypothesis was that the provision of clinically relevant data ahead of the assessment would enable clinicians to better prepare their assessment and create efficiency in their management of the clinical assessment, further enabling them to arrive at accurate clinical conclusions. To test this hypothesis, we investigated only patients referred through the AI self-referral tool, comparing patients who had provided clinical information in their referral to those who provided no clinical information.

First, we ensured that the patient groups did not differ with respect to the most relevant characteristics. Indeed, the groups did not differ with respect to severity (Mann-Whitney *U* test; *P*=.17), age (Mann-Whitney *U* test; *P*=.42), gender (Mann-Whitney *U* test; *P*=.44), ethnicity (Mann-Whitney *U* test; *P*=.39), disability status (Mann-Whitney *U* test; *P*=.62), or previous mental health treatment (Mann-Whitney *U* test; *P*=.76). This finding indicated that the groups were largely comparable. Nevertheless, we included these variables as covariates in the following analyses to ensure that even subtle differences were controlled for.

For the group in which additional clinical information was provided (mean assessment time 40.6 minutes), the clinical assessment required, on average, 12.3 minutes less time compared with the group without clinical information (mean assessment time 52.8 minutes). This effect was statistically significant (*t*_21,545_=−16.16; *P*<.001; Multimedia Appendix 3), and this could not be explained by differences in severity or demographics because the effect remained significant after controlling for these factors (*P*<.001).

Furthermore, in the group of patients with clinical information, the wait time for clinical assessment was shorter (mean 15 days) than that in the group without clinical information (mean 20.2 days).

This effect represented an average reduction of wait time of 5.2 days and was statistically significant (*t*_21,545_=−9.7; *P*<.001; Multimedia Appendix 3) and could not be explained by differences in severity or demographics because the effect remained significant after controlling for these factors (*P*<.001).

Finally, in the group with clinical information (recovery rate=58.7%), the recovery rates were significantly higher (*t*_5990_=2.3; *P*=.02; Multimedia Appendix 3) than in the group without clinical information (recovery rate=46.9%). This effect could not be explained by differences in severity, demographics, dropout rates, assessment and wait times, or by changes in treatment allocation because the effect remained significant after controlling for these factors (*P*=.03).

Notably, there were also some effects that seemed not to be driven by the clinical information provided ahead of time. There were no significant differences between patients with and without clinical information regarding dropout rates (*P*=.26), wait time for treatment (*P*=.51), and allocation to the accurate treatment level (*P*=.86). This finding suggests that the use of an AI self-referral solution improves access and treatment, with some of its effects being specific to the provision of high-quality symptom data to a clinician.

### Qualitative Analysis of the Reasons to Provide Clinical Information

We compared patients who provided all clinical information with those who did not provide this information to evaluate the impact of this clinical information on treatment outcomes.

However, because this study was a quasi-experimental setup, we aimed to understand why the patients chose to provide clinical information or not. To do so, we analyzed qualitative user research with 32 ex-patients to understand their experience with the AI self-referral tool using reflexive thematic analysis techniques. In this analysis, we focused on topics related to clinical information, with 2 relevant recurring topics identified. First, 38% (12/32) of the patients reported that the number of questions was perceived as long and potentially overwhelming. Second, 25% (8/32) of the patients reported that the nature of the clinical questions was emotionally difficult and could feel too heavy to complete.

This finding indicates that one of the main reasons for not providing clinical information might be time constraints and the feeling of being overwhelmed by providing detailed clinical information during referral.

However, the participants were ex-patients who were not seeking to refer themselves to treatment at the point of the study, which might make the collection of this information less directly relevant to them. Moreover, it is important to note that this study focused on the potential weaknesses of tool design. These results can be complemented by an analysis of 42,332 patients providing qualitative feedback after using the AI-referral tool in a real-world setting reported by Habicht et al [35]. In that analysis, 89% of the patients reported positive feedback on tool use, whereas only 7% gave neutral feedback, and 4% gave negative feedback. Notably, none of the negative feedback categories included complaints regarding the length or emotional content of the questions. This finding indicates that problems with the number of questions and their emotional content are rare in a real-world setting and might be more apparent when participants are pressed to suggest potential improvements. This finding is in line with the small number of patients not providing clinical information in our study.

## Discussion

### Principal Findings

In this study, we investigated the effects of implementing an AI self-referral tool in referral and assessment processes for mental health care. To this end, we compared patients referred through this AI tool against those referred through other means of referral within the same NHS Talking Therapy services and in a comparable time frame. In doing so, we demonstrated the improved service efficiency and clinical efficacy associated with this novel tool. Moreover, we investigated the mechanism through which these improvements were achieved and found that the provision of clinical information ahead of the mental health assessment was critical for many of the observed effects.

We found that patients accessing care through the AI tool showed reduced time required to complete their clinician-led assessment, reduced wait times for the assessment and treatment sessions, reduced dropout rates, improved accuracy of treatment allocation, and improved recovery rates. Moreover, we showed that the reduced assessment times, reduced wait times for assessment, and increased recovery rates were largely driven by the additional clinically relevant information collected from patients during their referral via the AI tool. Although our chatbot was friendly but not optimized to express compassion, the increase in efficiency can be seen as compassion for patients’ time and resources [36]. Although the effect of clinical information is more straightforward to explain for assessment time and recovery rates, we also observed an effect on wait times, which might appear less intuitive. The likely reason for this effect is a direct booking feature in the AI-referral tool, in which patients can immediately book an appointment in the services’ patient management system. However, this feature is only available once patients have provided all clinical information (ie, at the end of the referral process) to allow simple triage and assignment to the appropriate type of assessment (eg, question 9 of the PHQ-9 is required to assess suicidal ideation and thus associated risk). Therefore, this feature is not available for patients who did not provide clinical information.

It is important to note that we conducted multiple control analyses to rule out confounding factors and to establish the independence of these observed effects. Importantly, the severity of cases could not explain the differences between people referred through the AI tool compared with standard referrals. This finding is particularly important because any difference in recovery rates could be expected to be driven by symptom severity; therefore, we have ensured that the improvement seen by the AI self-referral tool cannot be explained by symptom severity. Other potentially confounding factors (eg, users of a new AI solution may have been more motivated to engage in therapy than patients referred by their general practitioner) are beyond the scope of our analyses and cannot be conclusively ruled out. Nevertheless, other studies evaluating the AI self-referral tool (Limbic Access) have also shown overall positive effects on provider level [37], that is, showing that NHS Talking Therapy providers using this tool showed overall increased recovery rates compared with matched Talking Therapy providers not using the tool. If a selection bias was the explanation for the observed effects, this would suggest no overall improvement in treatment outcomes for providers using the tool. Thus, findings from this related study [37] make a selection bias highly unlikely as an explanation for the observed benefits of the AI tool.

A randomized controlled trial is the gold standard for further confirming the observed effects of this study. However, randomized controlled trials have their shortcomings because they are costly to run and, therefore, limit the available sample size. We chose our experimental design to allow us to investigate an unprecedentedly large sample, yielding high statistical power and excellent ecological validity for our findings. Moreover, because our comparison is based on referrals within the same NHS Talking Therapy service, representing multiple geographies, our findings are unlikely to be driven by differences in demographic variables or general factors, such as geography, and should, therefore, be transferred to other Talking Therapy services.

In addition, we carefully tested whether all the observed effects were independent of each other. All reported effects remained significant when controlling for mutual influences, indicating that using the AI tool in the referral process has beneficial effects on all the variables reported in this study.

We investigated the mechanisms by which the AI self-referral tool improves clinical efficiency. We demonstrated that the provision of clinical information in referrals may be an important component of the observed effects. More specifically, we found that patients who provided clinical information during their referral had reduced assessment times, reduced wait times for assessment, and increased recovery rates. This finding indicates that the provision of clinical information ahead of clinical assessment could be a critical ingredient through which the AI tool achieved its effect on the tested outcome measures. This finding was hypothesized and showed that an increased amount of relevant information for the preparation of the clinical assessment has beneficial effects on patients and IAPT services.

In contrast, it is notable that not all effects observed for the AI solution (compared with other means of referrals) appeared to be driven by the provision of clinical information ahead of the clinical assessment. This might be expected for some of these effects. For instance, the reduction in dropout rates might be driven more by an overall positive experience that patients have when engaging with a friendly chatbot for submitting a referral, independent of the clinical information provided. Similarly, reductions in wait time for treatment might be driven more by the general administrative burden and overall resource availability rather than the specific clinical information provided in the referral.

However, it is notable that the provision of clinical information did not seem to have a significant effect on the accuracy of treatment allocation. This effect would have been expected to benefit from clinical information ahead of the clinical assessment. Nevertheless, there are 2 points to be considered with respect to this finding. First, there were a small number of patients (153/21,568, 0.71%) who did not provide clinical information and finished their treatment in this study, which dramatically reduced the power of the analysis compared with the analysis looking at the general effects of the AI solution compared with standard pathway referrals. Therefore, the nonsignificant results could be partly explained by the noise in a small sample. Second, it is notable that although the clinical information provided in this version of the AI tool is useful for many aspects of the clinical assessment process, it is fairly generic, mainly covering information about depression, generalized anxiety, and functional impairment. Although this information is useful in allocating accurate resources in the assessment and in prioritizing severe cases, it only provides limited information about the more specific symptoms that the patient experiences. This is especially true when the patient is experiencing mental health problems that do not represent depression or generalized anxiety. Therefore, the provision of more tailored and specific information at the point of referral would likely yield better results and support improvements regarding the allocation of treatment pathways.

### Limitations

Although this study aimed to maximize ecological validity and power using a large sample real-world data set, this decision has some limitations. As discussed above, this study was an observational study using a quasi-experimental setup. This means that the participants were not randomly allocated to each study arm (ie, type of referral). Although a multitude of control analyses have been conducted to ensure that the observed effects were not confounded by different characteristics of the patients (eg, case severity), it is not possible to measure and control for all potential confounding factors. Therefore, there remains the possibility of confounding factors between the study arms.

Moreover, we investigated the effects of clinical information and the observed benefits of the AI-enabled referral tool. Further, this was investigated using a quasi-experimental setup, which could have led to some form of confounds, even though careful statistical control of different characteristics has been conducted. It is noteworthy that in a separate usability study, patients reported that the self-referral process can be long and emotionally difficult, indicating that patients not providing clinical information could have done so because of time constraints or emotional burden. It is possible that these characteristics (eg, reduced time capacity or difficulties in facing emotional topics) could interact with treatment success and could influence the observed effects, such as improved recovery rates. Although we controlled for many confounding factors, it was not possible to further control for these potential effects and to conclusively rule out this possibility.

### Conclusions

This study represents, to the best of our knowledge, first evidence of the real-world impact of an AI-enabled self-referral tool in mental healthcare. The study was conducted with a large sample of patients in a mental health care setting, yielding a high ecological validity of the reported findings.

Notably, the results indicated a strong positive real-world impact of this novel AI tool (Limbic Access) on clinical efficacy and efficiency.

The setup for this study was quasi-experimental, so that not all confounding factors could be controlled completely. However, we assessed and controlled for the most relevant factors that could have differed between the groups of comparison. Notably, none of these factors could explain the observed effects, and all the effects remained significant after controlling for these factors.

It is critical to note that we provided converging evidence from multiple sources of data and different analyses. We conducted multiple control analyses to derive the most reliable and robust conclusions. Nevertheless, as none of the analyses included a randomized controlled trial, the possibility of confounding factors remained even though we controlled for most factors. Notwithstanding, the different analyses had different strengths and weaknesses, and no confounding factors could explain all the observed results.

Therefore, the results highlight the specific, beneficial role that well-designed AI solutions can play in augmenting the work of human clinicians by supporting elements of clinical work and through this, freeing up time for clinicians. This means that AI solutions can enable mental health care providers to deal with increased demand, even within a challenging funding environment that precludes increases in staffing levels.

## Appendix

Multimedia Appendix 1 Demographic characteristics of patients using the artificial intelligence (AI)-enabled referral tool and those who did not use the AI tool (ie, referred through other means). Although for patients using the AI tool, data were collected during the self-referral process, there were no individual-level demographics available for patients who did not use the AI tool. Group-level demographics were acquired using data from the National Health Service Digital database.

Multimedia Appendix 2 Comparison of treatment outcomes between referrals through the artificial intelligence (AI) self-referral tool versus standard referrals: (A) assessment time (in min), (B) wait time from referral to assessment (in days), (C) wait time from referral to first treatment session (in days), (D) dropout rates from treatment, (E) change in treatment level (measured as stepups and stepdowns in treatment level), and (F) recovery rate (ie, reliable recovery). Error bars indicate SEs. Because of the large sample size, some SEs are very small and thus hard to see. ∗∗∗*P*<.001.

Multimedia Appendix 3 Comparison of treatment outcomes between artificial intelligence tool referrals with and without clinical information: (A) assessment time (in min), (B) wait time from referral to assessment (in days), and (C) recovery rate (ie, reliable recovery). Error bars indicate SEs. Because of the large sample size, some SEs are very small and thus hard to see. ****P*<.001 and **P*<.05.

## Abbreviations

AI: artificial intelligence
IAPT: Improving Access to Psychological Therapies
NHS: National Health Service
PHQ-9: Patient Health Questionnaire-9

## Data and Code Availability

All analyses were based on sensitive clinical data, which we are not permitted to share with third parties. Summary statistics, as displayed in figures and text, are available upon request from the corresponding author.
