# Correction: Using Conversational AI to Facilitate Mental Health Assessments and Improve Clinical Efficiency Within Psychotherapy Services: Real-World Observational Study

**DOI:** 10.2196/57869

**Published:** 2024-03-12

**Authors:** Max Rollwage, Johanna Habicht, Keno Juechems, Ben Carrington, Sruthi Viswanathan, Mona Stylianou, Tobias U Hauser, Ross Harper

**Affiliations:** 1 Limbic Limited London United Kingdom; 2 Everyturn Mental Health Gosforth United Kingdom; 3 Max Planck University College London Centre for Computational Psychiatry and Ageing Research University College London London United Kingdom; 4 Department of Psychiatry and Psychotherapy Medical School and University Hospital Eberhard Karls University of Tübingen Tubingen Germany; 5 German Center for Mental Health (DZPG) Tubingen Germany

In “Using Conversational AI to Facilitate Mental Health Assessments and Improve Clinical Efficiency Within Psychotherapy Services: Real-World Observational Study” (JMIR AI 2023;2:e44358) the authors noted one error.

One author, Sruthi Viswanathan, was inadvertently omitted from the authorship list in the original publication of the paper. Sruthi Viswanathan has now been added to the authorship of the published paper as the fifth author, with the degrees "BTech, MRes" and the following affiliation:

Limbic Limited, London, United Kingdom

In accordance, the Conflict of Interest statement has also been updated to include this author. The originally published statement appeared as follows:

MR, KJ, JH, BC, and RH are employed by Limbic Limited and hold shares in the company. TUH works as a paid consultant for Limbic Limited and holds shares in the company.

This statement has been corrected to:

MR, KJ, JH, BC, SV and RH are employed by Limbic Limited and hold shares in the company. TUH works as a paid consultant for Limbic Limited and holds shares in the company.

The correction will appear in the online version of the paper on the JMIR Publications website on March 12, 2024, together with the publication of this correction notice. Because this was made after submission to PubMed, PubMed Central, and other full-text repositories, the corrected article has also been resubmitted to those repositories.

