# Supplementary material for: Using Conversational AI to Facilitate Mental Health Assessments and Improve Clinical Efficiency Within Psychotherapy Services: Real-World Observational Study
Source: JMIR AI. 2023 Dec 13;2:e44358. doi: 10.2196/44358 (PMC11041479; doi:10.2196/44358)
Supplement: Multimedia Appendix 1 [file ai_v2i1e44358_app1.pdf]

	Category	AI-tool	No AI-tool
**Age**	Under 18	0.4%	0.3%
	18 to 25	24.2%	25.8%
	26 to 64	72.7%	68.9%
	65 to 74	2.0%	3.4%
	75 to 89	0.7%	1.7%
**Ethnicity**	White	90.5%	93.1%
	Black or Black British	2.3%	2.5%
	Asian or Asian British	3.2%	3.2%
	Mixed	2.9%	0.5%
	Other	1%	0.6%
**Gender**	Female	70.3%	69.2%
	Male	28.1%	30.8%
	Non-binary	1.6%	0.04%
**Disability**	Yes	13.6%	7.3%
	No	86.4%	92.7%

Supplementary Table 1. Demographic characteristics for patients using the AI-enabled referral tool and for patients who did not use the AI tool (i.e. referred through other means). While for patients using the AI tool, the data was collected during the self-referral process, there were no individual-level demographics available for patients who did not use the AI tool. The group-level demographics were acquired through data from the NHS digital database.
