# Supplementary figures and images for: Using Conversational AI to Facilitate Mental Health Assessments and Improve Clinical Efficiency Within Psychotherapy Services: Real-World Observational Study
Source: JMIR AI. 2023 Dec 13;2:e44358. doi: 10.2196/44358 (PMC11041479; doi:10.2196/44358)

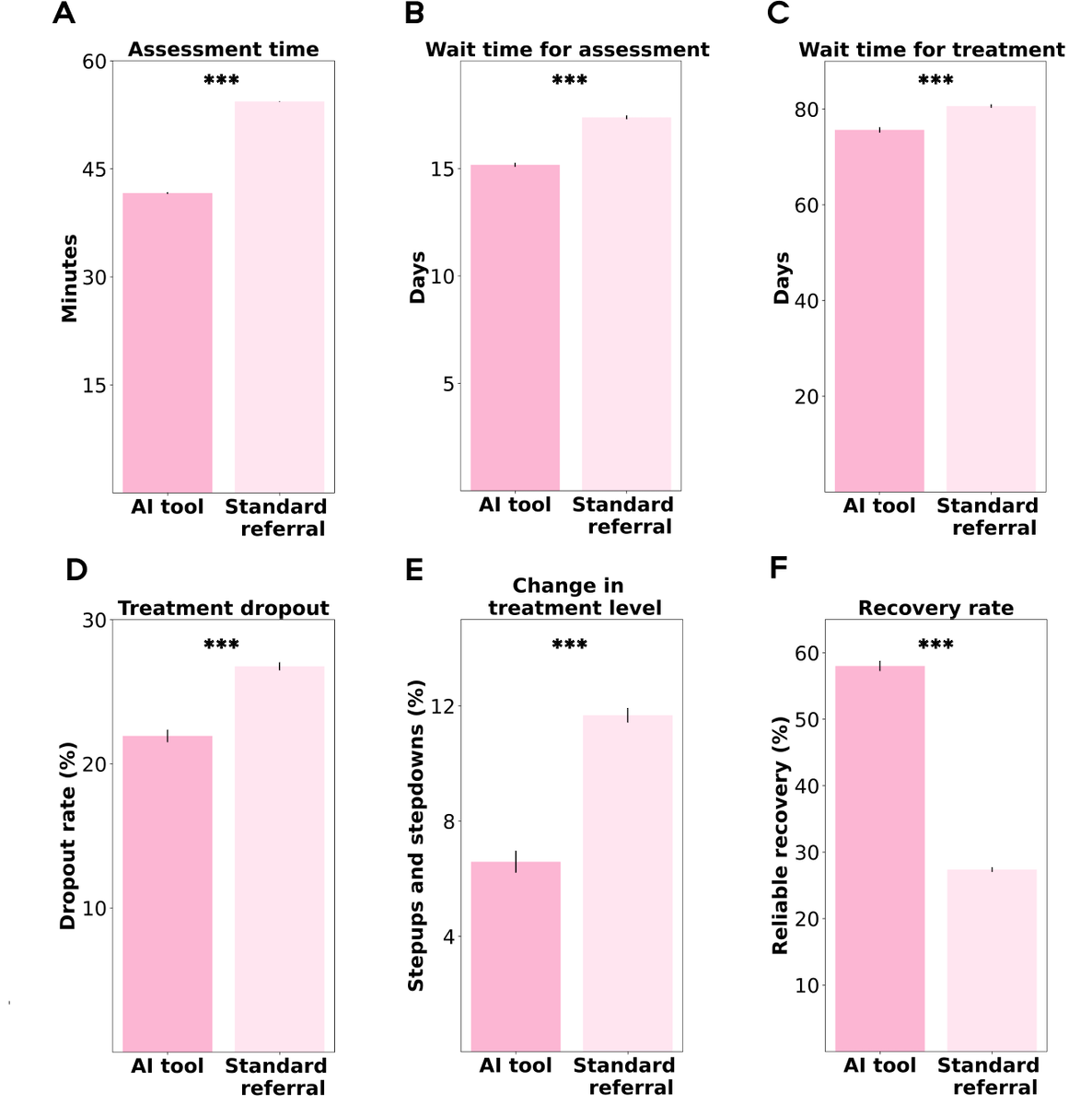

Supplement: Multimedia Appendix 2 [file ai_v2i1e44358_app2.png]

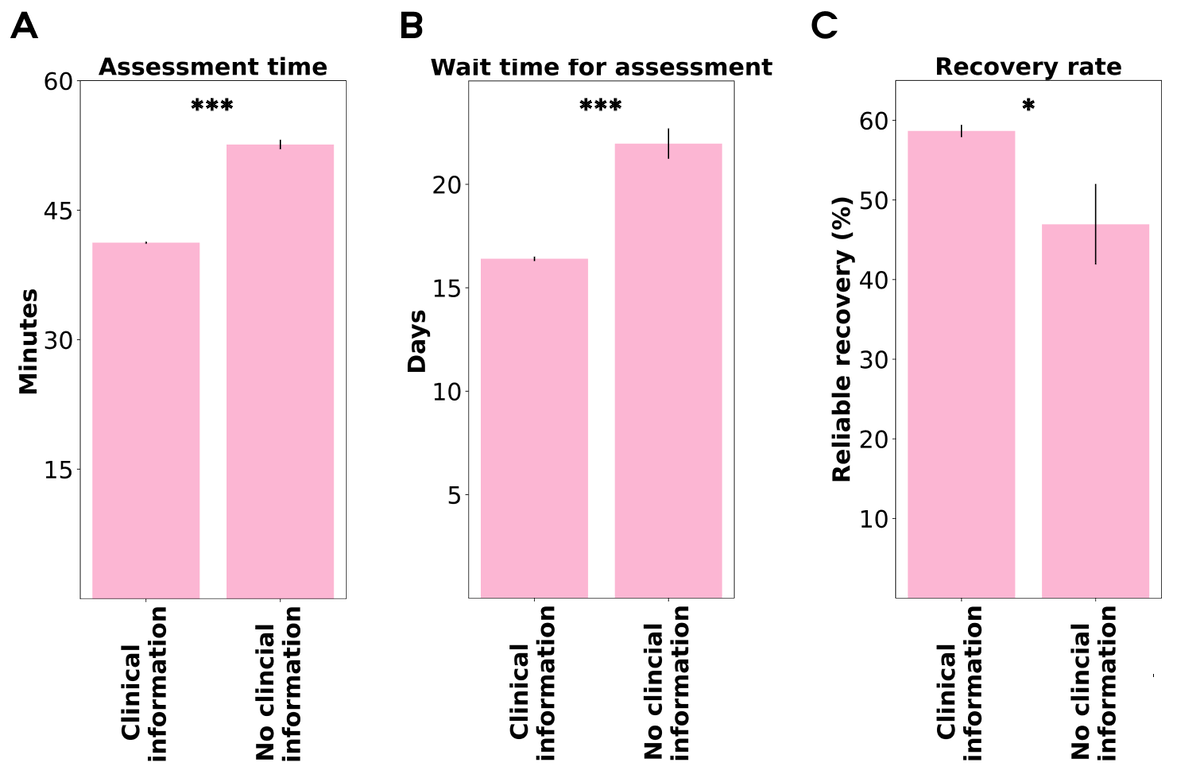

Supplement: Multimedia Appendix 3 [file ai_v2i1e44358_app3.png]
